# Engineering cell-fluorescent ion track hybrid detectors

**DOI:** 10.1186/1748-717X-8-141

**Published:** 2013-06-11

**Authors:** Martin Niklas, Steffen Greilich, Claudius Melzig, Mark S Akselrod, Jürgen Debus, Oliver Jäkel, Amir Abdollahi

**Affiliations:** 1 Division of Medical Physics in Radiation Oncology, German Cancer Research Center, INF 280, 69120 Heidelberg, Germany; 2 German Cancer Consortium (DKTK), National Center for Radiation Research in Oncology, Heidelberg Institute of Radiation Oncology, INF450/400, Heidelberg, Germany; 3 Molecular & Translational Radiation Oncology, Heidelberg Ion-Beam Therapy Center (HIT), University of Heidelberg Medical School and National Center for Tumor Diseases (NCT), German Cancer Research Center (DKFZ), 69120 Heidelberg, Germany; 4 Department of Radiation Oncology and Radiation Therapy, University Hospital Heidelberg, INF 400, 69120 Heideberg, Germany; 5 Heidelberg Ion-Beam Therapy Center (HIT), Im Neuenheimer Feld 450, 69120 Heidelberg, Germany; 6 Stillwater Crystal Growth Division, Landauer Inc., 723 1/2 Eastgate, Stillwater Oklahoma 74074, USA; 7 Center of Cancer Systems Biology, Nasa Specialized Center Of Research (NSCOR), St. Elizabeth’s Medical Center, Tufts University School of Medicine, Boston, MA, USA

## Abstract

**Background:**

The lack of sensitive biocompatible particle track detectors has so far limited parallel detection of physical energy deposition and biological response. Fluorescent nuclear track detectors (FNTDs) based on Al_2_O_3_:C,Mg single crystals combined with confocal laser scanning microscopy (CLSM) provide 3D information on ion tracks with a resolution limited by light diffraction. Here we report the development of next generation cell-fluorescent ion track hybrid detectors (Cell-Fit-HD).

**Methods:**

The biocompatibility of FNTDs was tested using six different cell lines, i.e. human non-small cell lung carcinoma (A549), glioblastoma (U87), androgen independent prostate cancer (PC3), epidermoid cancer (A431) and murine (VmDk) glioma SMA-560. To evaluate cell adherence, viability and conformal coverage of the crystals different seeding densities and alternative coating with extracellular matrix (fibronectin) was tested. Carbon irradiation was performed in Bragg peak (initial 270.55 MeV *u*^−1^). A series of cell compartment specific fluorescence stains including nuclear (HOECHST), membrane (Glut-1), cytoplasm (Calcein AM, CM-DiI) were tested on Cell-Fit-HDs and a single CLSM was employed to co-detect the physical (crystal) as well as the biological (cell layer) information.

**Results:**

The FNTD provides a biocompatible surface. Among the cells tested, A549 cells formed the most uniform, viable, tightly packed epithelial like monolayer. The ion track information was not compromised in Cell-Fit-HD as compared to the FNTD alone. Neither cell coating and culturing, nor additional staining procedures affected the properties of the FNTD surface to detect ion tracks. Standard immunofluorescence and live staining procedures could be employed to co-register cell biology and ion track information.

**Conclusions:**

The Cell-Fit-Hybrid Detector system is a promising platform for a multitude of studies linking biological response to energy deposition at high level of optical microscopy resolution.

## Background

To better understand the molecular mechanisms governing the radiobiological effects of particle therapy or space radiation simultaneous detection of cellular response and physical energy deposition is desired. Moreover, the growing field of particle therapy using protons and heavier ions (e.g. carbon) urgently needs novel means of biological dosimetry to accurately account for difference in relative biological effectiveness (RBE) [1], as a function of a plethora of particle variables such as location of ion traversal, ion type, energy and linear energy transfer (LET).

Earlier approaches to correlate physical radiation parameters with biological endpoints like cell survival (e.g. BIOSTACK experiment [2,3] and cell-coated CR39 detectors [4]) were hindered by a limited set of physical parameters that could be obtained. In addition, inability to autoclave the detector before cell deposition hindered the sterile cell cover.

**Figure 1:**
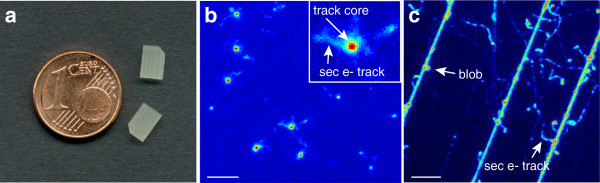
**Fluorescent nuclear track detector (FNTD). (a)** Two FNTDs (8 × 4 × 0.5 mm^3^). Courtesy of M.S. Akselrod, Landauer Crystal Growth Division. **(b)** Fluorescent image of the 270.55 MeV u ^−1^ carbon-ion tracks propagating perpendicular to the FNTD crystal surface. The brightest spots (physical energy deposition events) are attributed to carbon ions with a full width half maximum (FWHM) of approximately 500 nm [9]. The smaller, less intense spots to less densely ionizing particles like protons. The small structures around the carbon ion tracks arise from secondary electrons (sec e-) [10]. Insert: magnification of a single track spot. The track core is encoded by dark red. Scale bar, 5 *μ*m. **(c)** FNTD image after carbon irradiation (initial 270.5 MeV u ^−1^) parallel to the polished crystal surface. Secondary electron structures (small trajectories branching from the ion track) are visible. Instead of homogeneous energy deposition discrete blobs (bright spots) occur along the particle tracks. This is an illustration of stochastic nature of energy deposition along the heavy charged particle track. Courtesy of F. Lauer. Scale bar, 5 *μ*m.

Here we devise a novel strategy to establish a cell-fluorescent ion track hybrid detector (Cell-Fit-HD) based on fluorescent nuclear track detectors (FNTDs, Figure 1a). FNTDs based on Al_2_O_3_:C,Mg single crystals provide almost 100% detection efficiency of ion tracks imaged by confocal laser scanning microscope (CLSM) [5,6]. 3D information on energy deposition of ion tracks was obtained with diffraction-limited resolution and a multitude of physical parameters derived, such as location, direction, and possibly particle type and LET [6-9].

To develop the Cell-Fit-HD, the Al_2_O_3_:C,Mg crystal was autoclaved and coated with different human and murine cell lines to form a cell-crystal hybrid sample. We report here the successful development of protocols to grow human and murine cells on FNTDs to create a viable cell layer. To evaluate potential spectrum of applications for Cell-Fit-HD, we successfully tested different staining and fixation protocols for co-detection of different biological compartments and radiation particles parameters. Here, we demonstrate the feasibility of sequential read-out of the physical and biological information using CLSM without removing the cell layer from the Al_2_O_3_:C,Mg single crystal. The Cell-Fit-HD technology may provide a novel tool for of spatial correlation between biological readouts and single ion traversals.

## Methods

### **Al**_2_**O**_3_**:C,Mg based FNTDs**

The FNTDs are made of aluminum oxide single crystals doped with magnesium and carbon ions (*α*- Al_2_O_3_:C,Mg). The fluorescent color centers in these crystals are $F_{2}^{2+}(2\text{Mg})$-centers. They are the aggregate defects formed by two oxygen vacancies and two Mg-ion impurities [6]. Pristine $F_{2}^{2+}(2\text{Mg})$ color centers can be excited at 435 nm optical absorption band and emit fluorescent light at 520 nm. By exposing the detector to ionizing radiation, secondary electrons are generated, captured by the defects and transform them into $F_{2}^{+}(2\text{Mg})$ color centers. The radiation-transformed color centers have different optical properties: they absorb light in a band centered at 620 nm light, prompting fast 750 nm fluorescence. The intensity of this radiation-induced 750 nm fluorescence depends on local energy deposition of ionizing radiation. Accordingly, FNTDs allow for high spatial resolution particle track visualization by confocal microscopy [6] (Figure 1b,c) and subsequent 3D particle track reconstruction [7,11]. The system is sensitive for ions with LET > 0.5 keV *μm*^−1^[5,8]. FNTD offers a detection efficiency close to 100% for the entire spectrum of primary particles and fragments at energies found in ion beam cancer therapy [5]. The current limit of maximum accessible track fluence is in the range of 5 · 10^7^ c*m*^−2^[5], relevant to fluences providing clinical doses.

### Cell cultures and characterization

All cells and Cell-Fit-HD were cultured in humidified atmosphere under standard culture conditions (37°C, 5% C*O*_2_). Human lung adenocarcinoma epithelial (A549) and human glioblastoma (U87) cells were cultured in Dulbecco’s modified Eagle medium (DMEM, Biochrom AG, Berlin, Germany, Cat. No. FG 0415). Human epidermoid carcinoma (A431) and human prostate cancer (PC3) cells were cultured in Roswell Park Memorial Institute (RPMI) 1640 medium (Biochrom AG, Cat. No. FG 1215). Murine astrocytoma (SMA-560) cells were cultured in DMEM/Ham’s F-12 liquid medium (Biochrom AG, Cat. No. FG 4815). All culture media were supplemented with 10% Fetal Bovine Serum (FBS, Biochrom AG, Cat. No. S 0615) and 1% penicillin- streptomycin.

PC3, A549 and A431 cells were obtained from Deutsche Sammlung von Mikroorganismen und Zellkulturen (DSMZ, Braunschweig, Germany), U87 cells were obtained from American Type Culture Collection (ATCC) and SMA-560 was kindly donated by Dr. Wolfgang Wick, Clinical Cooperation Unit Neurooncology, German Cancer Research Center, Heidelberg, Germany.

### Cell-coating of FNTDs

FNTDs were autoclaved and washed in Dulbecco’s Phosphate-Buffered Saline (DPBS). For direct coating each FNTD was placed in a well of a multi-well plate (24 Well Cell Culture Multiwell Plate, CELLSTAR ^*Ⓡ*^, Greiner Bio-One) and the polished surface was covered with cells at different plating densities, from 20·10^3^, 100·10^3^, 300·10^3^ to 400·10^3^ m*l*^−1^, 0.5 ml per well. The Cell-Fit-HDs were kept in humidified atmosphere until a confluent monolayer had developed. The medium was changed the first time after 24 h and then in 48 h intervals and the cell layer was tightly monitored using a wide-field microscope equipped with phase contrast filter (Axiovert 40C, Zeiss). To test the feasibility of FNTD surface coating with extracellular matrix components, FNTDs were placed in a multiwell plate filled with fibronectin (Bovine Fibronectin, without Bovine Serum Albumin, R&D Systems, Minneapolis, USA) at different concentrations (1, 2.5, 5, 10 or 20 ng *l*^−1^, diluted with DPBS) and incubated for 50 min at humidified atmosphere. After incubation FNTDs were gently washed twice with DPBS and covered with cells. Pre-coated FNTDs were sonicated for 15 min and washed with DPBS to remove any solid leftovers before autoclaving them for reuse.

### Cell labeling and immunofluorescence staining

To test whether the FNTD, as a substrate, interferes with standard fixation and staining procedures, the cell layer of the hybrid detector was labeled with a series of dyes.

Cells were labeled with a fluorescent dye often used in life cell imaging, Cell-tracker CM-DiI Molecular Probe ^*Ⓡ*^, Cat. No. C700) at a concentration of 1.5 *μ*g *l*^−1^ in DPBS (1 ml) for 8 min at humidified atmosphere first and then for additional 15 min at room temperature (RT). After labeling, cells were gently washed twice with DPBS. Finally, the Cell-Fit-HD was placed in fresh cell culture medium. For imaging of CM-DiI Leica DM IL LED inverted widefield-microscope equipped with a mercury lamp, CY3 green filter cube and a CCD camera (Leica DFC420 C) were used.

To test viability, A549 cells were stained with 10^−3^ mol *m*^−3^ calcein acetoxymethly ester (Calcein AM, Molecular Probes ^*Ⓡ*^, Cat. No. C1430). Prior to staining the cell layer was washed with DPBS. After incubation (RT) for 30 min, cells were gently washed with DPBS. For Calcein AM imaging the inverted CLSM 710, Confocor 3 (Zeiss) was equipped with 488 nm Argon laser line, EC Plan-Neofluar 40x/1.30 oil objective, main beam splitter (MBS) 488 nm and PMT detection (detection window: 493- 617 nm). As Calcein was the single dye detected, there was no potential spectral overlap within this window. In case of multiple dyes the window could be narrowed around the emission peak at 513 nm.

For membrane and nuclear dual staining, glucose transporter (Glut1) specific antibody and a standard immunofluorescence (IF) protocol were employed. A549 cells were fixed with 4% paraformaldehyde (PFA) in PBS for 10 min (RT). Cells were washed with PBS. After fixation, cells were permeabilized with 0.1% Triton X-100 in PBS (10 min, RT), washed with PBS, blocked in PBS containing 2% bovine serum albumin (BSA) (30 min, RT), and washed with PBS again. Glut1 specific primary antibody (Abcam ^*Ⓡ*^, Cat. No. ab652) was used in a dilution of 1:200 in 1% BSA in PBS with 1 h incubation at RT. After the washing step with PBS containing 1% BSA secondary antibody Alexa Fluor 555 goat anti-mouse IgG conjugate (Molecular Probes ^*Ⓡ*^, Cat. No. A-21422) was used in dilution of 1:1000 in 1% BSA/ PBS by incubating for 1 h at RT. Cells were washed with PBS containing 1% BSA. For nuclear counter staining, cells were incubated for 5 min (at RT) in HOECHST 33342 (Molecular Probes ^*Ⓡ*^, Cat. No. H1399)/ PBS solution with a final concentration of 2 *μ*g ml^−1^ and washed with PBS. The CLSM 710 Confocor 3 equipped with an EC Plan-Neofluar 40x/1.30 oil objective was used for imaging of the Glut1/HOECHST dual staining. We used the 405 nm diode laser line for HOECHST 33342 and 561 nm Argon laser line for Alexa Fluor 555. The MBS 458/561 nm and MBS 405 nm were used for detection of Alexa Fluor 555 and HOECHST 33342 respectively. The detection window for emitted fluorescent light of Alexa Fluor 555 and HOECHST 33342 were 566-697 nm and 410-550 nm respectively.

For nuclear staining after irradiation of the Cell-Fit-HD, the cells were fixed and stained with HOECHST 33342, 15 min post irradiation. For the fixation and IF procedure the same protocol as for the Glut1/HOECHST dual staining was used.

For imaging with CLSM 710, Confocor 3, the Cell-Fit-HD was placed in uncoated glass bottom culture dishes (MatTek Corp., Ashland, USA, Cat. No. P35G-1.5-10-C). Zeiss Immersol ^TM^518 F was used as an immersion medium. Fluoromount-G ^TM^ (SouthernBiotech, Birmingham, USA) was used as a mounting medium for all IF staining except HOECHST 33342 single stain. For HOECHST 33342 single stain PBS was used. For live imaging (Calcein AM, CM-DiI) cell culture medium was used as mounting medium.

### Irradiation setup for cell-coated FNTD

Carbon ion irradiations of Cell-Fit-HD were performed with the therapy beam of the Heidelberg Ion Beam Therapy Center (HIT) at Heidelberg University Hospital. The ion beam fluence was adjusted to 1.5·10^6^ cm^−2^ using controls of the treatment system, resulting in an average of 1.3 hits per nucleus (assuming all nuclei to be of equivalent size with an area of 10 x 10 *μm*^2^). In total, a 12 x 12 cm^2^ field was irradiated homogeneously using raster scanning with a pencil beam of 10.1 mm in diameter (full width at half maximum) and a distance of 2 mm between two raster spots. Approximately 60,000 particles were delivered in each spot. The Bragg peak was extended by using a 3 mm Ripple filter. The cell layer was placed in the rising flank of the Bragg peak (initial carbon ion energy of 270.55 MeV u ^−1^, corresponding equivalent range in water $r_{H_{2}O}$= 13.70 cm). As stopping material 11.7 cm of PMMA with $r_{H_{2}O}$= 13.57 cm was placed in front of a multiwall plate (24 Well Cell Culture Multiwell Plate, CELLSTAR ^*Ⓡ*^, Greiner Bio-One) containing the cell-coated FNTDs (see Additional file 1: Figure S1). The multiwell plate was placed perpendicular to the incident beam. Irradiation setup in experimental room at HIT with horizontal beam propagation requires vertical positioning of samples. The FNTD crystals are 0.5 mm thick with equivalent range in water $r_{H_{2}O}$= 1.65 mm. The back side of the FNTDs without cell coating were facing the incident beam and were attached to the bottom (polystyrene with $r_{H_{2}O}$= 1.2 mm) of the multiwell plate by small agarose droplets. The air gap between the culture well and the PMMA was not considered in the total $r_{H_{2}O}$. Culture wells were filled with cell culture medium to keep the cell coating viable during irradiation. The total amount of materials in front of the iso center (vacuum exit window, beam application monitoring system, air) corresponds to a $r_{H_{2}O}$ of 2.89 mm.

### Microscopy settings for sequential read-out of cell-coated FNTD

For the sequential read-out of the FNTD component and the cell layer of the Cell-Fit-HD, we used the CLSM 710, Confocor 3 equipped a 63x/1.45 NA oil objective, APD/PMT/T-PMT detection.

For the FNTD read-out we used the protocol as described in [10]. The FNTD crystals were scanned by the 633 nm Helium Neon laser line (100% transmission). A main dichroic beam splitter (MBS) 488/561/633 nm was used to separate the emission signal from the excitation light. A 655 nm long-pass filter was used in fluorescent emission path and the Avalanche Photo Diode (APD) for emission detection was used in photon counting mode. The microscope detector pinhole aperture was set to 1 Airy disk diameter unit (AU). The cell layer was imaged with 405 nm diode laser line (30 mW, 4.0% transmission) for HOECHST 33342. For the photomultiplier (PMT) detection of HOECHST 33342, a MBS 405 nm was used (detection window 410 nm - 495 nm). For the FNTD and cell layer acquisition, we limited the line-scanning repetition to 4 and a pixel dwell time to 2.80 *μ*s. In both cases, the size of the imaging field was 134.784 x 134.784 *μ*m^2^ with a total number of 1152 x 1152 pixels and a pixel size of 0.117 x 0.117 *μ*m^2^ (estimated according to Rayleigh criterion). This results in a frame-acquisition time of 14.9 s. The acquired FNTD image stack was adjusted to cover an axial range of about 120 *μ*m (measured from the detector surface) with a z-interval (*Δ*z) of 3 *μ*m. This allows for accurate single ion track reconstruction as described in [11]. The acquired cell layer stack covered a range of about 10 *μ*m (*Δ*z= 0.3 *μ*m).

## Results

### FNTD crystals are biocompatible

To develop Cell-Fit Hybrid Detectors, first the adherence, viability and growth of different human (A549, A541, U87, PC3) and murine (SMA-560) cell lines on the FNTD surface were tested. We were able to produce a viable and stable cell layer on the polished crystal surface. Cells were seeded directly or after coating with fibronectin as an intermediate layer to enhance cell adhesion on the crystal surface (Table 1). We varied the plating density and fibronectin concentration to optimize the coating and to quantify cell proliferation kinetics. SMA-560 (with fibronectin), PC3 (without fibronectin), A431 (with/without fibronectin) and A549 (with/without fibronectin) cells were able to form a uniform coating after 24 h using high seeding density (≥ 300,000 ml ^−1^). In comparison, the proliferation rate of U87 cells (300,000 ml ^−1^, with/without fibronectin) was found to be lower. After 120 h these cells still formed a loosely packed network. For A549 cell line, a uniform cell coating were also be achieved after longer incubation time (after 48 h) using lower plating density (100,000 ml ^−1^).

**Table 1:** Cell-coating experiments

**Cell line**	**Seeding density [1/well]**	**Fibronectin [ng/ml]**	**Cell-coating**		
			**> 24 h**	**> 48 h**	**> 120 h**
SMA-560	10 000	2.5, 5, 10, 20	Single cells, no network	Single cells, no network (*)	Network of overlapping cells
	150 000	2.5, 5, 10, 20	network of overlapping cells		
	150 000	-	-	-	-
U87	10 000	2.5, 5, 10, 20	single cells, no network	single cells, no network (*)	single cells, no network
	150 000	2.5, 5, 10, 20	loosely packed network		
	150 000	-	loosely packed network		
PC3	200 000	2.5	-	-	-
	200 000	-	tightly packed monolayer		
A431	200 000	2.5	tightly packed monolayer		
	200 000	-	tightly packed monolayer		
A549	50 000	-	-	tightly packed monolayer	
	150 000	1, 2.5, 5	tightly packed monolayer		
	150 000	-	tightly packed monolayer		

The table shows the results of cell coating on the surface of FNTD crystals using different cell lines with and without a fibronectin intermediate layer. Each well contained 500 *μ*l. Cell culture medium was exchanged every 48 h. (*) Proliferation rate is lower compared to cells grown on the untreated bottom of a culture well.

At low seeding concentration (20,000 ml ^−1^) the proliferation rate of SMA-560 (with fibronectin) and U87 cells (with fibronectin) was decreased as compared to cells growing on the cell culture treated surface, e.g. on the bottom of the wellplates in which the Cell-Fit-HDs were incubated. SMA-560 (with fibronectin) developed a network of overlapping cells on the FNTD’s surface after 120 h. Lower concentrations of fibronectin (2.5, 5 ng ml ^−1^) seemed to be favorable for both cell lines using low seeding concentrations.

**Figure 2:**
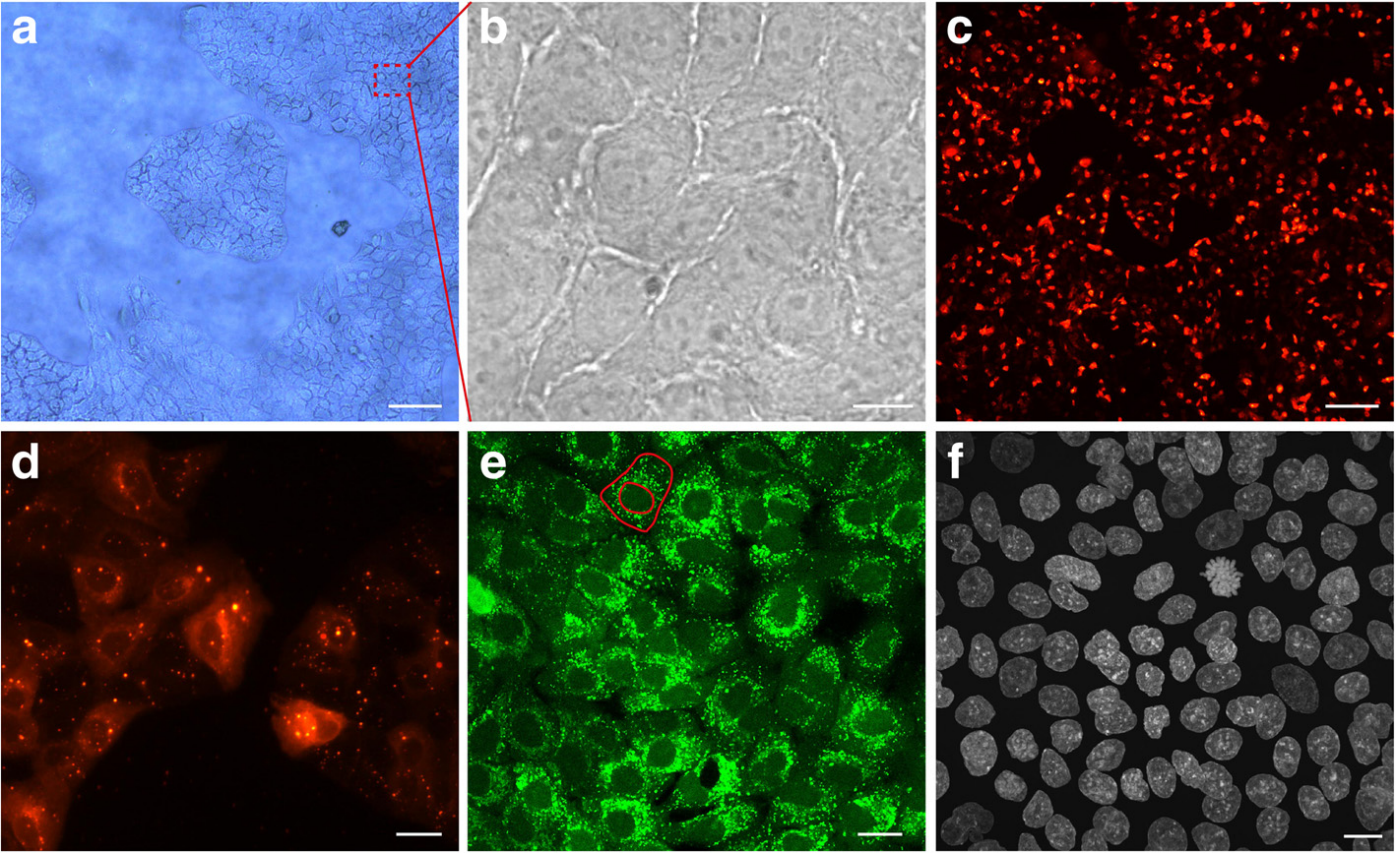
**A549 cell coating. (a)** A549 cells cultured on the FNTD crystal surface starting to form a confluent monolayer. A typical island formation with branching cells is clearly visible. Scale bar, approximately 200 *μ*m **(b)** Magnified section of a confluent monolayer. The cells are tightly packed. It is difficult to contrast cells from the transparent crystal substrate with light microscopy. Scale bar, 10 *μ*m. **(c)** CM-DiI labeled and proliferating cells forming a confluent monolayer. Scale bar, approximately 200 *μ*m **(d)** Section of CM-DiI labeled monolayer reveals clear cytoplasmic coloring. Cytoplasmic granules (multilamellar bodies) exhibit a strong fluorescent signal. Scale bar, approximately 20 *μ*m. **(e)** Cell layer is labeled with Calcein AM to test cell viability. The outer red line indicates the cell membrane. The cell nucleus is defined by the inner red line. A strong perinuclear fluorescent signal with many bright spots (cytoplasmic organelles) and round nuclei indicate good cell viability. Scale bar, 20 *μ*m. **(f)** Immunofluorescent labeling of cell nuclei by HOECHST 33342 stain. A uniform monolayer of proliferating cells is visible. Scale bar, 10 *μ*m. Images **(a)**, **(c)**, and **(d)** were obtained by wide field microscopy whereas images (b, e, and f) were obtained in confocal fluorescent mode. Images **(a)**-**(e)** show live cell stainings. In **(f)** cells are fixed with 4% PFA.

Concerning the morphology, all cells were spread and flat. They exhibited the same shape as grown on standard cell culture treated surfaces under controlled conditions (incubator, humid atmosphere). In general, the epithelial tumor cell lines, A549, A431 and PC3 formed a tightly packed monolayer in comparison with gliomas SMA-560 and U87 developing networks of overlapping cells. Figure 2a,b shows a section of a monolayer of A549 cells without additional fibronectin coating. Those cells exhibited a cubic morphology.

### Cell labeling and immunofluorescence staining of Cell-Fit-HD

To evaluate the feasibility of co-detection of biological information using fluorescence based cell stains on the surface of FNTDs, we applied various cell labeling and immunofluorescence protocols on Cell-Fit-HDs. We found that the FNTD crystal surface as a substrate is compatible with all staining protocols tested. Calcein AM labeling was used for live cell imaging and for determining the viability of A549 cells (Figure 2e). The high levels of fluorescence signal as well as the shape of the cell nuclei indicated good cell viability. The bright spots are organelles (multilamellar bodies) in the cytoplasm with high esterase activity correlating with active metabolism.

We used CM-DiI labeling as cell tracker dye to achieve a better contrast between cells and the crystal substrate (Figure 2c,d). This labeling is based on thiol-reactive chloromethyl moiety that allows the dye to covalently bind to cellular thiols. CM-DiI staining suggested exocytotic activity of cells as small bodies with fluorescent signal are present in intercellular space.

**Figure 3:**
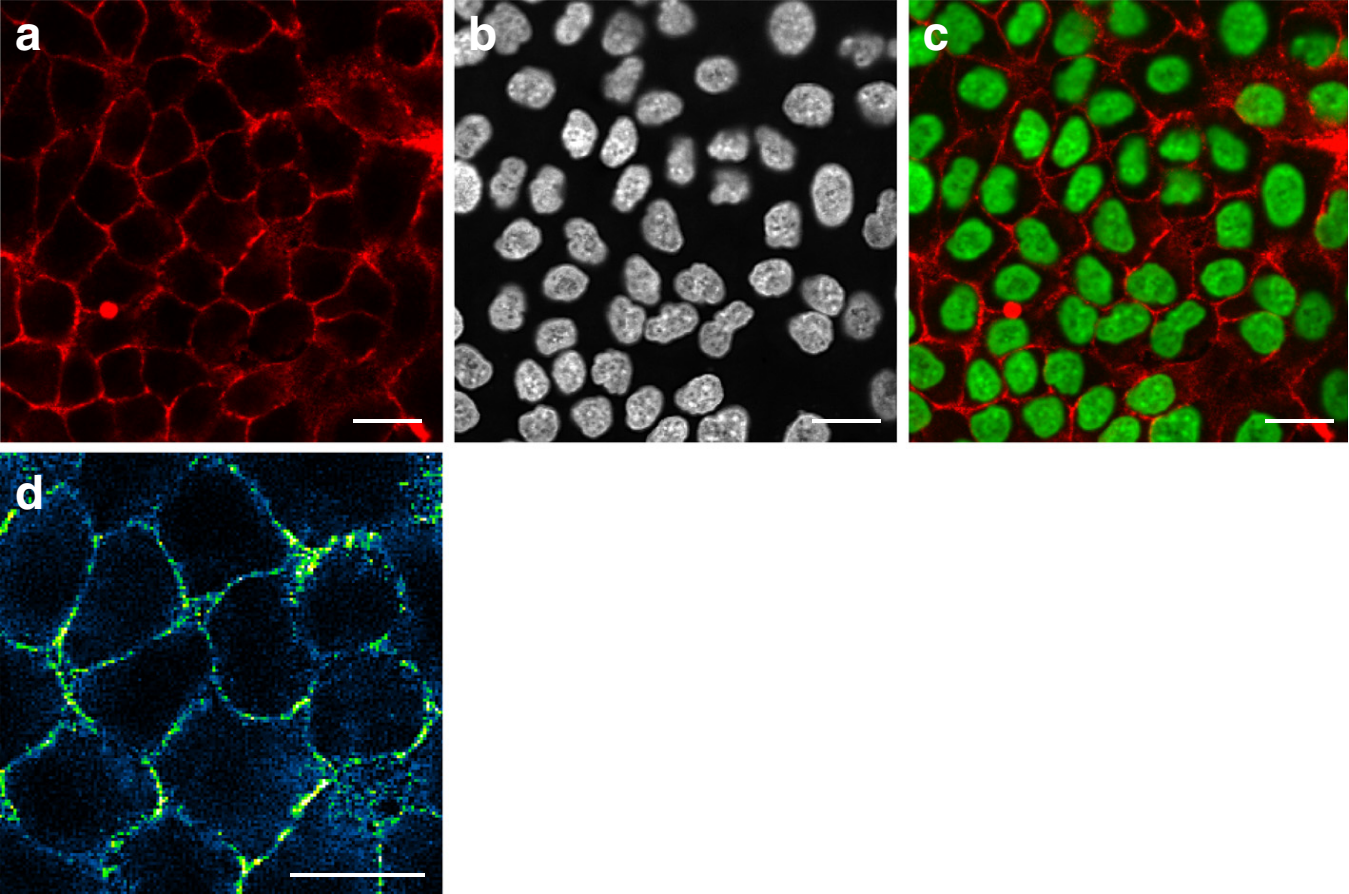
**Membrane and nuclear staining of A549 Cell-Fit-HD. (a)** Glucose transporter Glut1 staining visualizes the A549 cell membrane. Glut1 is mainly accumulated at the membrane. The diffuse cytoplasmic signal may arise from permeabilisation during immunofluorescent staining. **(b)** HOECHST staining as nuclear counterstain. **(c)** Merging of Glut1 and HOECHST images. A549 cells form a tightly packed monolayer with strong cell-cell adhesion. **(d)** A section of **(a)** with different color coding (blue-green-yellow) is shown. The distinct yellow bright spots indicate a discrete strong accumulation of Glut1 at the membrane. Scale bars, 20 *μ*m. **(a)**-**(d)** were obtained by confocal fluorescent microscopy.

To estimate the cell density of tightly packed A549 coating we did immunohistochemical analysis and used HOECHST as nuclear stain (Figure 2f). The bulk had a cell density of approximately 6.3·10^5^ cm ^−2^. We combined HOECHST with Glut-1 staining to visualize cell membranes and to detect cell-cell adhesion (Figure 3). There is strong accumulation of Glut1 at the membrane thus forming a sharply bounded network. Together, membrane labeling with Glut1 or CM DiI demonstrated that A549 cells maintained their tight epithelial cell-cell contact in Cell-Fit-HD.

### Irradiation of Cell-Fit-HD and sequential read-out

Placing the A549 cell-coated FNTD in a well filled with culture medium allows for an easy handling and mounting for the irradiation (Additional file 1: Figure S1). The cells stayed alive during irradiation. The total irradiation time (adjusting the ion-beam fluence to an average of 1.3 hits per nucleus) was less than 10 s.

**Figure 4:**
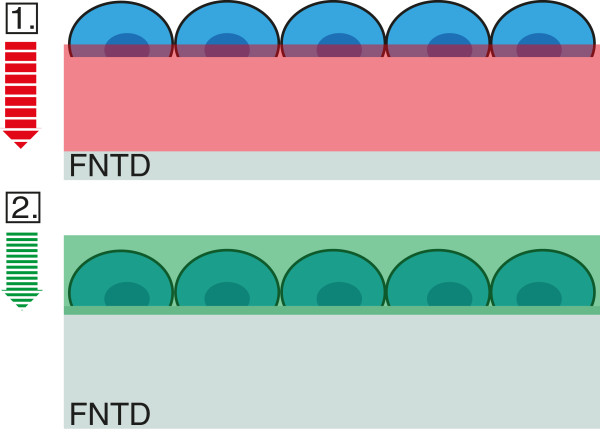
**Sequential read-out of Cell-Fit-HD.** To avoid photoionization of FNTD crystals the imaging is first performed with the red laser on FNTD crystal and only after that the scan continues with blue laser on stained cell layer. The total axial range for the acquired image stack is about 120 *μ*m with depth increment (*Δ*z) of 3 *μ*m for FNTD crystal and *Δ*z= 0.3 *μ*m for the cell-layer. The axial range for the image stack of cells is about 10 *μ*m. The difference in optical sectioning is indicated by the striped arrows.

After conventional fixation (using 4% PFA) and staining procedure for HOECHST (cells remained on FNTD) the detector and the cell layer were imaged successfully in two consecutive steps using the same CLSM (Figure 4). No post-irradiation chemical processing of the FNTD or removal of the cell layer was necessary.

When imaging the cell layer prior to the FNTD, an increase in the detector background signal in the surface region (first few *μ*m) occur. We adjusted the scan and detection parameters (see Methods) of the FNTD read-out to achieve relatively fast acquisition (frame-acquisition time of 14.9 s) without forfeiting much signal-to-noise ratio (SNR).

**Figure 5:**
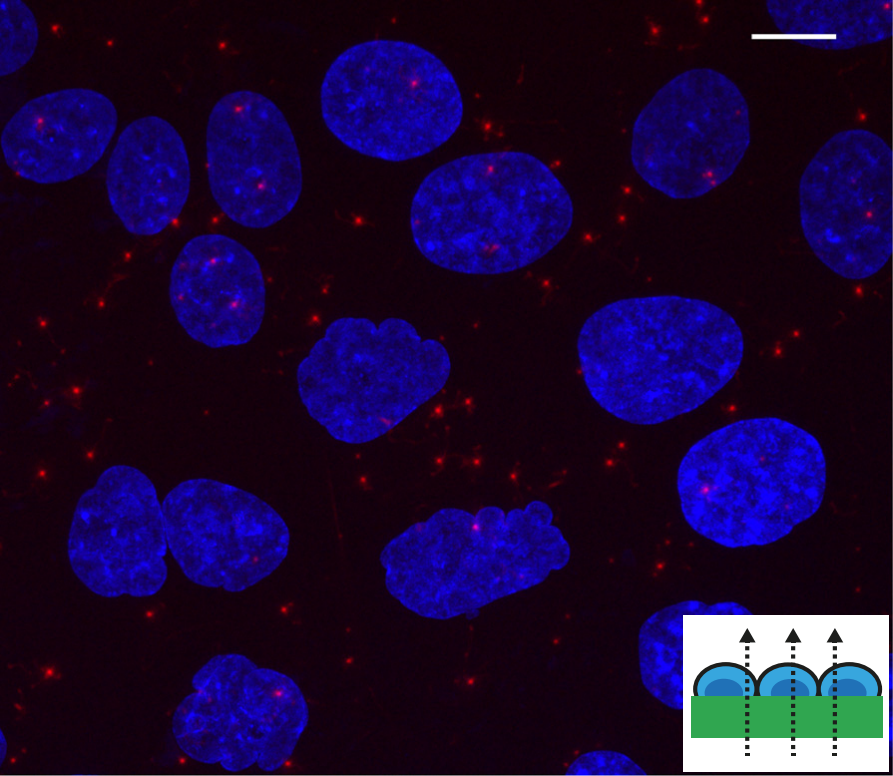
**Correlation between carbon ion tracks and A549 cell layer.** Superposition of cellular response data (maximum intensity z-projection) with an image of the acquired FNTD image stack. The red spots are the ion tracks (with FWHM of approximately 500 nm [9]). The small trajectories branching from the ion tracks are tracks of secondary electrons in the FNTD crystal. The cell nuclei (depicted in blue) are labeled with HOECHST. The surface of cell-coated FNTD was set perpendicular to the incident carbon ion beam (small insert). Initial carbon ion energy was 270.55 MeV u ^−1^ and fluence - 10^6^ cm ^−2^. Scale bar, 10 *μ*m.

Figure 5 shows the spatial correlation between carbon ion tracks and A549 cell layer gained after the sequential read-out. Compared to cell nuclei (diameter ≈10 *μ*m) single ion tracks visualized by track spots - the characteristic signature left in the FNTD crystal after irradiation - have a diameter of less than 1 *μ*m.

## Discussion

To our knowledge, we report here the first successful development of a hybrid detector technology, Cell–Fit–HD, allowing simultaneous acquisition and correlation of radiation track information with cellular and molecular parameters. It was recently shown that Al_2_O_3_:C,Mg single crystal based fluorescent nuclear track detectors (FNTDs) allow for visualization and characterization of single particle tracks by conventional fluorescence microscopy [7,11]. Given that confocal microscopy is a common method for detection of molecular- and cellular biology parameters, it was hypothesized that development of hybrid detectors will provide a hitherto unmatched correlation of physical with biological parameters at high resolution, only limited by light diffraction [7]. However, little is known about the properties of the polished and planar detector surface with regard to their biocompatibility. Based on the relatively inert chemical reactivity of Al_2_O_3_:C,Mg crystals and their high thermal resistance (up to 600°C) [12] we were able to successfully autoclave the crystals, as a prerequisite for sterile cell coverage. The ability of the FNTD technology to precisely measure energy deposition along carbon ion tracks was neither affected by autoclaving nor by prolonged exposure to high humidity under standard culture growth conditions. Next, we tested the adherence, the viability and conformal coverage of the crystals coated with a panel of human and murine cells.

We found that these crystals are biocompatible for all of the cell lines tested enabling the development of Cell-Fit-HD. The cells were spread and flat thus indicating good cell attachment. Cell adhesion could also benefit from the doping of Al_2_O_3_ with magnesium ions as several molecules involved in cell adhesion such as integrins, have binding sites for divalent ions [13]. In addition the use of different extracellular matrix (ECM) component to enhance cell adhesion, as performed in this study, treatment of the crystal surface with plasma or additional bombardment of the detector surface with Mg ^2+^ ions [14] may further enhance cell adhesion. The crystal surface was suitable for most of the cell lines tested in order to gain a viable and uniform cell coating (Table 1). It was possible to achieve uniform cell coating (Table 1) either in short incubation time (after 24 h) using high plating density (≥ 300,000 ml ^−1^) or longer incubation time using a lower plating density. For the two brain tumor cell lines, human U87 and murine SMA-560 at low seeding concentrations (20,000 ml ^−1^) the fibronectin concentration could further be optimized to improve cell coating or another extra cellular matrix component like collagen could be tested. The fibronectin layer has a height of several nm and does not interfere with the limited mechanical working distance of a microscope objective at high magnification.

As compared to standard treated surfaces for cell cultures all tested cell lines maintained their morphology when plated on the crystal surface. Based on the cell types the tested cell lines formed either glioma-like cell networks (SMA-560 and U87) or tightly packed epithelial monolayers (PC3, A431, A549). The evaluation using conventional phase contrast microscopy was hindered by the transparency of Al_2_O_3_:C,Mg (Figure 2a). Our data indicated that neither the physical properties of the crystal component nor the physiological properties of the cell lines were compromised.

We selected A549 cells for subsequent Cell-Fit-HD studies due to their superior performance in terms of cell adhesion, viability, proliferation, metabolism and formation of a tightly packed epithelial network on the crystal surface without the need of additional ECM coverage. Al_2_O_3_:C,Mg crystal proved to be compatible with standard fixing and staining procedures. The fluorescent dyes did not accumulate at the surface nor seemed to affect the surface properties, hence causing low background during image acquisition of the cell layer. Moreover, fixation (4% PFA) did not distort A549 cell morphology when seeded on the detector surface (Figure 2). The cells can be read-out in situ on the crystal surface by conventional confocal microscopy. When reading out the cell layer prior to the crystal with blue (405 nm) laser light, it is possible to photoionize pristine $F_{2}^{2+}(2\text{Mg})$ color centers in the surface region by two-photon absorption processes resulting in increase in background 750 nm fluorescence used for track imaging. To avoid this increase in background signal, we imaged the crystal with red (633 nm) laser scanning prior to the cell layer scanning with the blue laser. The crystal read-out with the red laser in turn does not bleach fluorescent dyes in the cell layer nor seems to affect the cell layer in another manner (e.g., morphology). No post-irradiation chemical processing of the crystal or removal of the cell layer is necessary for the sequential read-out, thus eliminating a significant source of error. The high cell density (A549) with strong cell-cell adhesion (Figure 3) and fixation directly after irradiation limits cell migration and distortion of the actual spatial correlation or hit statistics.

To examine the capability of Cell-Fit-HD to detect biological processes governing radiation effects in different cell compartments with physical energy deposition along ion tracks, visualization of different key cellular compartments was evaluated. Our data indicate that Cell-Fit-HD is compatible with standard immunofluorescent techniques. Therefore Cell-Fit-HD may be used to detect radiation induced molecular events on cell membrane such as differential regulation of cell adhesion molecules/receptors and their downstream signaling events as demonstrated by membrane staining using antibody (Glut1) or life dyes (CM DiI). Moreover, direct damaging effects of ionizing irradiation in different cellular compartment such as mitochondria or nucleus could be tested by specific staining of these compartments as demonstrated here for DNA-staining in nucleus (HOECHST) or candidate alternative dyes such as DAPI or ToPro. Together, application of standard fluorescent staining techniques on the cell layer and the sequential read-out of the Cell-Fit-HD enable spatial correlation between single ion traversal and cell biology (Figure 5). Currently, algorithms are developed to correlate the physical information achieved in crystal compartment with biological events detected in the cell layer.

## Conclusions

Development of a next generation cell-fluorescent ion track hybrid detector (Cell-Fit-HD) is reported permitting co-detection and direct correlation of physical and biological information at high resolution using standard fluorescence microscopy. Detection of physical energy deposition along carbon ion-tracks is based on Al_2_O_3_:C,Mg crystals (FNTDs). We showed that these crystals are chemically inert, could be autoclaved and provide a biocompatible surface for prolonged culture of different cell lines. The crystals provided a substrate for a uniform cell coating. Cells maintained their morphology, metabolism, proliferated and remained viable for a long observation period in Cell-Fit-HD. Moreover, the quality of the FNTD readout was not compromised by different standard fixation procedures and labeling of different cell compartments using life fluorescent dyes or immunofluorescence. This offers the possibility of carrying out a multitude of live-imaging and fluorescent staining experiments directly linking the effect of physical energy deposition along ion tracks with subsequent cellular events on membrane, cytoplasm, organelles or nuclear compartment. The here reported method for development of Cell-Fit-HD will be instrumental for introduction of a novel generation of compact detectors facilitating research in radiobiology laboratories with limited access to more complex detector technologies and microbeam delivery systems. Cell-Fit-HD can be irradiated with different radiation qualities, i.e. protons and heavier ions.

## Competing interests

The authors declare that they have no competing financial interests.

## Authors’ contributions

MN performed experiments, analyzed data and together with AA wrote the manuscript. CM participated in the cell-coating experiments. MA developed the crystal material and FNTD imaging technique. MN and SG developed the co-detection of physical- and biological parameters by confocal microscopy. SG initiated the project. MN, SG, JD, OJ and AA designed experiments and interpreted the data. All authors edited the paper. All authors read and approved the final manuscript.

## Authors’ information

Martin Niklas and Steffen Greilich shared first authorship.

## Supplementary Material

Additional file 1 **Figure S1.** Irradiation setup for Cell-Fit-HD. The Cell-Fit-HD was irradiated perpendicular to the incident carbon ion beam. Irradiation setup at Heidelberg Ion-Beam Therapy Center (HIT) requires a vertical positioning of the sample. Only a single well of a multiwell plate is shown.The FNTD is attached by agarose droplets to the polystyrene bottom of the multiwell plate. The well is filled with culture medium to keep the cells viable during irradiation. It is sealed with Parafilm^*Ⓡ*^ M (Pechiney Plastic Packaging). The air gap between PMMA block and the multiwell plate is neglected as the corresponding energy loss is very small. The range in water ($r_{H_{2}O}$) of all elements in the beam path (position and fluence monitors) between exit window of the beam line and isocenter of the incident ion beam is 2.89 mm. Click here for file
