# Supplementary figures and images for: Engineering cell-fluorescent ion track hybrid detectors
Source: Radiat Oncol. 2013 Jun 11;8:141. doi: 10.1186/1748-717X-8-141 (PMC3699405; doi:10.1186/1748-717X-8-141)

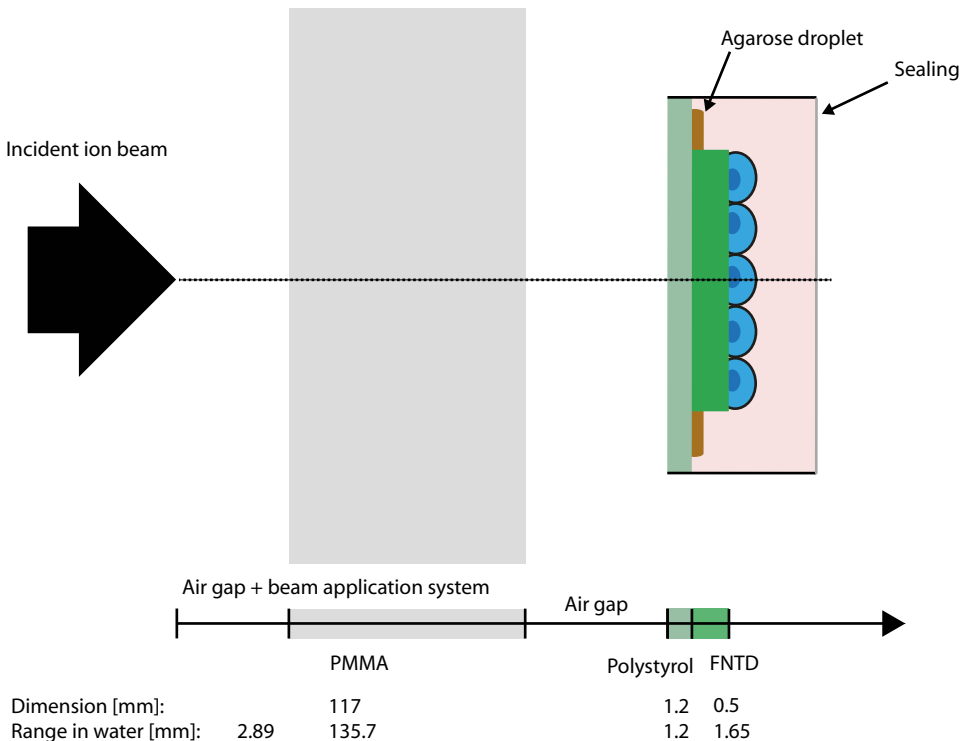

Supplement: Additional file 1 — Figure S1. Irradiation setup for Cell-Fit-HD. The Cell-Fit-HD was irradiated perpendicular to the incident carbon ion beam. Irradiation setup at Heidelberg Ion-Beam Therapy Center (HIT) requires a vertical positioning of the sample. Only a single well of a multiwell plate is shown.The FNTD is attached by agarose droplets to the polystyrene bottom of the multiwell plate. The well is filled with culture medium to keep the cells viable during irradiation. It is sealed with ParafilmⓇ M (Pechiney Plastic Packaging). The air gap between PMMA block and the multiwell plate is neglected as the corresponding energy loss is very small. The range in water (rH2O) of all elements in the beam path (position and fluence monitors) between exit window of the beam line and isocenter of the incident ion beam is 2.89 mm. [file 1748-717X-8-141-S1.pdf]
